# Protocol for the effect evaluation of Individual Placement and Support (IPS): a randomized controlled multicenter trial of IPS versus treatment as usual for patients with moderate to severe mental illness in Norway

**DOI:** 10.1186/s12888-014-0307-7

**Published:** 2014-11-18

**Authors:** Vigdis Sveinsdottir, Camilla Løvvik, Tonje Fyhn, Karin Monstad, Kari Ludvigsen, Simon Øverland, Silje Endresen Reme

**Affiliations:** Uni Research Health, Uni Research, POB 7810, Bergen, NO-5020 Norway; Uni Rokkan Centre, Uni Research, Nygårdsgaten 5, Bergen, NO-5015 Norway; Department of Public Mental Health, Division of Mental Health, Norwegian Institute of Public Health, Nydalen, POB 4404, Oslo, NO-0403 Norway; Department of Psychosocial Science, University of Bergen, POB 7807, Bergen, NO-5020 Norway

**Keywords:** Disability, Individual placement and support, IPS, Mental health, Quality of life, Randomized controlled trial, Vocational rehabilitation, Return to work, Labor market participation, Supported employment

## Abstract

**Background:**

Roughly one third of disability pensions in Norway are issued for mental and behavioral disorders, and vocational rehabilitation offered to this group has traditionally been dominated by train-and-place approaches with assisted or sheltered employment. Based on a more innovative place-and-train approach, Individual Placement and Support (IPS) involves supported employment in real-life competitive work settings, and has shown great promise for patients with severe mental illness.

**Methods/design:**

The study is a multicenter Randomized Controlled Trial (RCT) of IPS in a Norwegian context, involving an effect evaluation, a process evaluation, and a cost/benefit analysis. IPS will be compared to high quality treatment as usual (TAU), with labor market participation and educational activity at 12 months post inclusion as the primary outcome. The primary outcome will be measured using register data, and the project will also include complete follow-up up to 4 years after inclusion for long-term outcome data. Secondary outcomes include mental health status, disability and quality of life, collected through survey questionnaires at baseline, and after 6 and 12 months. Participants will include patients undergoing treatment for moderate to severe mental illness who are either unemployed or on sickness or social benefits. The estimated total sample size of 400–500 will be randomly assigned to the interventions. To be eligible, participants must have an expressed desire to work, and sufficient Norwegian reading and writing skills to fill out the questionnaires.

**Discussion:**

The Effect Evaluation of Individual Placement and Support (IPS) will be one of the largest randomized controlled trials to date investigating the effectiveness of IPS on competitive employment, and the first study to evaluate the effectiveness of IPS for patients with moderate to severe mental illness within a Norwegian context.

**Trial registration:**

Clinicaltrials.gov: NCT01964092. Registered October 16th, 2013.

## Background

Roughly one third of disability pensions in Norway are issued for mental and behavioral disorders [1]. The proportion is even higher among those awarded disability pension before the age of 40, where such disorders account for 58% of all awards [1]. While approximately 65% of people with severe mental illness want to work, only 15% are employed, suggesting a large gap of unmet needs [2]. There are several economic, social, and moral arguments for facilitating work participation and preventing exclusion from working life among people suffering from mental illness, and there is strong evidence suggesting that work can have beneficial effects on physical and mental health [3,4]. Concerns that competitive employment could be harmful for patients with severe mental illness are widespread, but emerging evidence has shown that work participation improves both clinical and social functioning, finances, self-esteem, and psychiatric symptoms for these patients [5–8]. These observations can be understood through different models. While a social selection hypothesis states that good health is a condition for employment, evidence shows stronger support for a social causation hypothesis where employment leads to improved health [4]. Job loss and unemployment is strongly associated with mental illness, while re-employment has been shown to reverse the negative effects of unemployment on mental health [4,9,10]. The relationship between employment and health may be partly explained by mechanisms of health behaviors such as alcohol and substance abuse, diet, physical activity, and smoking [11–13]. A Norwegian study of alcohol disorders and re-employment suggested that although some selection to unemployment does occur, the high prevalence of harmful drinking among the unemployed is mainly a result of the unemployment rather than vice versa, and that reducing unemployment could reduce alcohol problems in Norway [14].

The Norwegian welfare system provides financial aid and security for individuals unable to work. It is however important to optimize the services provided to help people to remain in working life despite episodes of health problems or periodical troubles with functioning at work.

Vocational rehabilitation and training approaches generally fall within two traditions, *train and place* or *place and train*. In the former approach, the client undergoes targeted training in an adapted or sheltered environment to acquire necessary skills, before attempting to enter competitive employment in an arena relevant to the training. At present, this tradition dominates vocational rehabilitation in Norway, with programs such as Work with assistance (AB) and Traineeship in a sheltered business (APS).

In contrast, the place and train principle represents a novel approach where the primary goal is direct employment in real-life settings, without any preceding training. Individual Placement and Support (IPS) is a prime example of a model within this tradition, where the goal is to provide professional services to help people with disabilities participate in the competitive labor market [15]. The intervention is manualized, and integrates supported employment as a component of psychological treatment rather than a separate service. It focuses on the clients’ preferences, with the philosophy that anyone who wants to work can find a regular job in the community, and that no one should be deprived of that opportunity [16–20]. A further characteristic of the IPS model is that clients are not screened for work readiness, but rather on expressed desire to work. It does not involve intermediate work experiences, transitional employment or sheltered workshops, but actively facilitates job acquisition and provides ongoing support once the client is employed. The model has got strong empirical support in the American context, with positive results in terms of both vocational and non-vocational outcomes for people with severe mental illness [21–29], especially for young adults [30]. There is also favorable evidence for IPS from studies conducted in European countries, but these have generally not reached as strong results as the studies from the US [31–33]. The IPS transportability outside the US thus remains unanswered [33]. As of yet, no studies have evaluated the effectiveness of IPS in Norway. The Norwegian context is characterized by high job security, low unemployment, and a generous welfare system, which stands out from most other contexts where IPS has been evaluated [34]. Furthermore, previous studies have all included people with severe mental illness, with no attempts to expand the target group to involve people with moderate mental illness as well.

## Methods/design

### Aims and objectives

The main aim of the study is to evaluate the effect of IPS compared to high quality TAU offered to people with moderate to severe mental illness in six IPS centers located in different Norwegian counties. We aim to address the following questions:

- Is IPS more effective than high quality TAU in terms of increasing labor market participation?
- Is IPS more effective than TAU in terms of improving mental health status and quality of life?
- Are there specific Norwegian policy factors that will influence the effectiveness of IPS?
- What are the challenges of implementing and disseminating IPS in a Norwegian context?
- Is IPS cost-effective compared to TAU?

### Outcome measures

The primary outcome of the study is *increased labor market participation in ordinary paid employment* or *education.* Employment and enrollment in education will be identified using register information, and will be analyzed separately, but also combined as a general indicator of increased activity on the pathways to work. Labor market participation will be operationalized by combining a) being registered in the Norwegian Labor and Welfare Administrations (NAV) State Register of Employers and Employees, and b) not receiving unemployment or sickness benefits, c) not receiving work assessment allowance or disability pension with a higher degree of disability than at study inclusion, and d) a registered annual income. This will align with the previous IPS literature, where competitive employment is defined as “permanent jobs paying commensurate wages in integrated community settings (i.e., employing nondisabled workers) and available to anyone (not just individuals with disabilities)” [33]. Educational activity will be operationalized as started or completed education as registered in the Education Statistics by Statistics Norway (SSB). To ensure comparability of results with previous IPS-studies [35], we will collect survey information to supplement the register data regarding measures of job acquisition (e.g. time from study entry to first job start), duration (e.g. cumulative numbers of weeks worked in all jobs), intensity (e.g. percentage working at least 20 hours a week), and productivity (e.g. total hours worked/wages).

The secondary outcomes, *mental health status, disability* and *quality of life*, will also be measured through survey questionnaires, in addition to questions on background and health-related variables:

- *Mental Health Status* will be measured using the 14-item Hospital Anxiety and Depression Scale (HADS), consisting of 2 subscales: anxiety (7 items) and depression (7 items) [36], and the 35-item Beliefs About Voices Questionnaire-Revised (BAVQ-R), consisting of 5 subscales: malevolence (6 items), benevolence (6 items), omnipotence (6 items), resistance (9 items), and engagement (8 items) [37].
- *Disability* will be measured using the 12-item version of the WHO Disability Assessment Schedule 2.0 (WHODAS 2.0), consisting of 6 different functioning domains: cognition (2 items), mobility (2 items), self-care (2 items), getting along (2 items), life activities (2 items), and participation (2 items) [38].
- *Health-related quality of life* will be measured using the health index from the EuroQol questionnaire (EQ-5D) [39].
- *Alcohol abuse* will be measured using the 3-item Alcohol Use Disorders Identification Test (AUDIT-C), screening test for heavy drinking and/or active alcohol abuse or dependence [40].
- *Drug abuse* will be measured using the Drug Use Disorders Identification Test (DUDIT), screening for drug use, drug-related problems, and/or drug dependence [41].
- *Subjective Health Complaints* will be measured by the 29-item Subjective Health Complaints Inventory (SHC), consisting of 5 subscales: musculoskeletal pain (8 items), pseudoneurology (7 items), gastrointestinal problems (7 items), allergy (5 items), and flu (2 items) [42].
- *Social support* will be measured using a revised 11-item version of the Social Support Inventory [43,44] using 2 subscales as suggested by Øyeflaten et al. [45]: directive social support (4 items) and nondirective social support (7 items).
- *Fatigue* will be measured by the 11-item Chalder Fatigue Questionnaire (CFQ) consisting of 2 subscales: physical fatigue (7 items) and mental fatigue (4 items) [46].

The questionnaire package was developed in collaboration with a client representative who provided input on the length, design and formulation of the information and questionnaires.

### Data collection

Survey data will be collected from both groups at baseline, 6 and 12 month follow-up. Baseline questionnaires will be administered and collected at the IPS centers by the person conducting the recruitment interviews. Data will be collected using iPads with secure survey software (Qualtrics®), but paper questionnaires will also be available. Electronic data will be collected in offline mode, before being connected to the Internet at the IPS center and sent to a secure online database. Paper questionnaires will be temporarily stored at the IPS center and forwarded to Uni Research Health every three months. Follow-up questionnaires will be administered electronically to participants who provide their e-mail address at baseline, or on paper via regular mail if this is preferred.

Using data from the national social insurance register and the national employee-register in Norway allows for complete and objective data to be collected every month for every participant, with no loss to follow-up. Data will be collected retrospectively 3 years before baseline, and prospectively 4 years after baseline [47]. This information will be collected for each month of follow-up, to examine sensitivity of the primary 12-months results. Additionally, the long follow-up ensures comparison with a recent Swedish IPS study, where effectiveness was first seen at 18 months [32].

### Study design

The project is designed as a randomized controlled trial (RCT) using mixed methods, and includes: 1) an effect evaluation, 2) a process evaluation, and 3) a cost/benefit-analysis.

The effect evaluation is the main focus of the project, accompanied by a cost/benefit analysis to provide an economic assessment of the effect. However, in order to explore different stakeholders’ experiences and various facilitating factors and barriers in the implementation of the project, there is also a need for a process evaluation involving both qualitative and quantitative data.

### Effect evaluation

The effect evaluation is conducted as a RCT, which is the gold standard for clinical trials and ideal for achieving our aim of *comparing the effect* of different interventions to increase labor market participation.

#### Interventions

Participants will be randomly allocated to one of two groups, receiving different active interventions that aim to increase labor market participation and prevent withdrawal from the labor market. One group will receive *Individual Placement and support (IPS)* at their local IPS center, focusing on employment in the competitive labor market. IPS is a structured approach based on eight principles; competitive employment, eligibility based on client choice, integration of rehabilitation and mental health services, attention to client preferences, personalized benefits counseling, rapid job search (starting within one month), systematic job development, and time-unlimited and individualized support [19]. A pure control group with no intervention was considered to be neither feasible nor legally or ethically acceptable in this study. The other group will therefore be referred from the IPS center to NAV to receive a high quality version of *treatment as usual (TAU)*. This involves being offered a prioritized spot in a vocational rehabilitation scheme, primarily Work with assistance (AB) and/or Traineeship in a sheltered business (APS). AB involves assistance by a personal facilitator, and includes finding suitable work, negotiating wage and employment conditions, modified duties, and follow-up at the work place. APS involves testing of work capability within a sheltered environment doing tasks that are modified to individual skills and challenges, with follow-up as necessary by an advisor. Participants in this group may also be offered additional interventions based on the individual needs, as they normally would in TAU.

#### Participants and recruitment

Eligible participants are patients currently undergoing treatment for moderate to severe mental illness, who are currently out of the labor market but have an expressed desire to work. They could be either unemployed, on sick leave benefits, or on other social benefits at the time of inclusion. Participants will primarily be recruited from the various District Psychiatric Centers (DPS), which are placed in secondary care, but could also be recruited from stepped-care initiatives located in primary care. All participants are or will be connected to a treatment-team linked to a job specialist (caseworker trained in IPS). We aim to recruit 200–250 participants in each group, with a total sample size of 400–500.

#### Sample size calculation

Our estimates of sample size are based on international input-data from previous IPS-studies. As a general comparison, the typical sample size in the previous IPS studies range from 41 to 312 participants [33]. The studies from countries most similar to the Norwegian context include Australia (n = 41), England (n = 188), the Netherlands (n = 151), and Sweden (n = 120) [32,33]. For the non-US studies, the mean competitive employment rate was 50% for IPS and 20% for controls [33]. The study showing the lowest effect of IPS to this date came from England, with competitive employment rates of 22% for IPS and 11% for controls [48].

**Figure 1:**
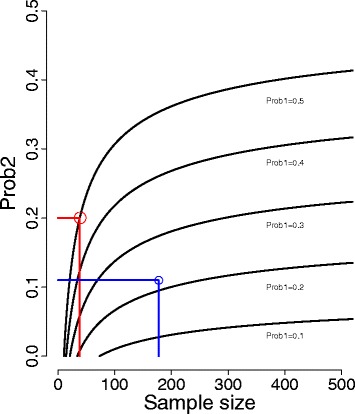
**Sample size calculation with possible rates.**

If we use 50% and 20% as possible rates, we will need 39 participants in each group in order to demonstrate a statistically significant difference (with a 5% significance level and power of 80%), and in a potential scenario with rates of 22% and 11%, we will need 178 participants in each group (Figure 1).

There is, however, reason to believe that the target group in the current project is more heterogeneous than in the international studies, involving people with both moderate and severe mental illness. Furthermore, we anticipate that the high quality treatment as usual will result in higher competitive employment rates in the control group than in the international studies, particularly compared to US studies. We therefore aim to recruit between 400 and 500 participants.

Secondary outcomes are measured with questionnaire data, and we expect an approximate response rate of no more than 60%. This may have consequences for the conclusions drawn from secondary outcome data. However, compared to a previous IPS study [22] that found a significant effect on quality of life (mean 5.4, SD = 1.9 vs 4.8, SD = 1.7), the same difference could be detected with 142 participants in each group with a power of 80%, which in this project should be feasible even with a response rate of 60%. The analyses of the secondary outcomes will be performed with inverse probability weights to account for possible attrition bias [49].

Sufficient inclusion and response rates are crucial for enabling robust main analyses and for the possibilities to document effects of IPS with statistical certainty, as well as for allowing additional subgroup analyses. Rates lower than the estimated total sample size of 400–500 will reduce the chance of detecting smaller effects that may be important on a larger scale, and will limit the possibilities to further investigate which groups may benefit most from the intervention.

#### Subgroups

Any significant main effects between the IPS intervention and control intervention group may allow for subgroup analyses. Regression analyses stratified on intervention group will additionally be conducted in order to study variables or sub-groups showing a stronger or weaker effect. The following a priori defined subgroups will be investigated: diagnostic groups (moderate vs. severe mental illness), work status at baseline (unemployed vs. disability benefits), and previous work history (previously employed vs. never been employed).

#### Inclusion and exclusion

Inclusion criteria involved not working (unemployed, on sick leave, or on social benefits), undergoing treatment for moderate to severe mental illness, having an expressed desire to work, and sufficient Norwegian reading and writing skills to be able to fill out the questionnaires.

#### Randomization

Upon receiving new eligible participants, trained and competent personnel at the IPS centers will perform an introductory interview with the participant, informing about the study, and asking the individual to fill out an informed consent form in order to be included in the study and receive an ID-number. When the participant has filled out the baseline-questionnaires, the person conducting the introductory interview contacts the research technician at Uni Research Health by email, stating the participants ID-number, gender, and year of birth. Using computer-generated randomization lists, the participant will be allocated to one of the two groups. A 2:1 randomization ratio will be applied the first months of recruitment to ensure that the IPS centers can run according to capacity. The randomization procedure will however strictly adhere to the formal requirements of adequate randomization at all times.

#### Statistical analyses

The analyses will follow the “intention to treat” principle. We will apply administrative register data from NAV and SSB in order to evaluate the primary outcome of the project; labor market participation in ordinary paid employment and educational activity. Beyond giving consent at inclusion, this part of the effect evaluation is not dependent upon participants’ willingness to respond to surveys, as register data on employment, education and use of social security benefits will be complete for all participants. Consequently, common problems related to self-report such as recall bias, justification bias, and attrition will be avoided for our primary outcomes. Randomization helps to assure that there are no systematic differences between the IPS intervention and control intervention groups at baseline, in terms of both observable and unobservable characteristics. The design allows for a comparison of proportions employed in each group over time before and after inclusion, including a test of whether differences are statistically significant. Additionally, we will perform regression analysis using logistic regression models in order to a) control for any remaining variation in observable characteristics between groups, b) investigate which factors, other than IPS intervention, have an impact on labor market participation, and c) analyze heterogeneity in the response to IPS. Groups will be compared on secondary outcome measures using survey data at baseline, and after 6 and 12 months.

### Process evaluation

The purpose of the process evaluation is to investigate the quality and accuracy of the implementation of the IPS intervention. Furthermore, the results of a process evaluation may provide indications for weaknesses either in the implementation of the program, or theoretical weaknesses, in case of unexpected findings in the effect evaluation. Investigating the implementation of key components of the intervention will provide context for the interpretation of main results, as well as highlight any need for improvements in future intervention design, methods, and the implementation process of the intervention. Together, the results of process investigation will help to determine the feasibility of the intervention.

Following the framework of Steckler & Linnan [50], supplemented by the broader process evaluation literature of Baranowski & Stables [51] and Green & Glasgow [52], the process evaluation will report on the following:

- *Reach*, referring to the participation rate of the target group and the representativeness of these [52], which affects the external validity of the study.
- *Dose delivered*, which refers to the amount of the intended intervention that is actually offered to the participants [50].
- *Dose received* may refer to the applicant’s use of teaching material, conducting home assignments or attending meetings [50].
- *Fidelity* refers to the providers’ compliance with the IPS protocol, which is a standardized measurement [53]. The fidelity ratings are conducted regularly throughout the project period.
- *Satisfaction* refers to the participants’ overall satisfaction with the program.
- *Perceived effectiveness* is the participants’ perception of the usefulness of IPS in obtaining employment.
- *Facilitators and barriers* will shed light on methodological or practical issues as well as factors in the local context that helps or hinders the implementation of the intervention.

The process evaluation will take place alongside the project implementation, and data will be collected throughout the project period in order to monitor how the intervention is established and adapted over time.

#### Quantitative material

The process evaluation includes collecting quantitative data that can describe how the different elements of the intervention were implemented. This information will be important in order to identify possible methodological challenges at the individual IPS centers, as expressed by the measures in the above framework: Reach, dose delivered and received, and fidelity. To explore local barriers towards successful implementation, we will also measure employers’ attitudes to hiring people who are at risk of being excluded from work life, as well as review characteristics of the local labor market.

1. **Questionnaire items at 6-month follow-up:** 6**–**8 items will be included in the questionnaire at 6 months, measuring dose received, satisfaction, perceived efficacy, and perceived barriers and facilitators. The items are constructed based on process evaluation theory and adapted to the conditions of the current intervention.
2. **Other data sources:** To determine the dose given, data will be collected from the job specialists’ register for each participant. To determine reach, the job specialists’ register of people who declined or accepted participation will be used. Publicly available statistical data will be used to examine the representativeness of the reached group in relation to the target group at large, using the variables age and gender.
3. **Fidelity measurements:** The IPS fidelity scale is an established measurement [53], and the ratings will be carried out by a partly independent team that is trained, certified, and experienced in making such measurements. The measurements will be performed regularly throughout the project period. The fidelity ratings will evaluate the competence and methodology in conducting the intervention, as well as provide information on the frequency of contact and interaction with the participants. The qualitative interviews included in the fidelity measurements will provide a greater understanding and description of how the criteria have been attained, or why they may not have been attained. Although the process evaluation largely focuses on the IPS intervention group, qualitative interviews will also be conducted with participants in the control intervention group to get an idea of what control measures the ordinary follow-up has consisted of.
4. **Questionnaire for employers:** The quality of the relationship between employer and employee can play an important role for commitment to the work place, job performance, and job satisfaction [54,55]. In order to examine attitudes towards hiring employees with various physical or mental illnesses and/or other limitations, researchers in the project group have developed a questionnaire that will be distributed to employers. In addition to providing a general overview of attitudes towards groups at risk of discrimination, we may also examine if any changes in attitudes occur after hiring employees with various challenges.
5. **Local labor market:** The local labor market is likely to vary between the centers’ catchment areas, and availability of work can influence on the outcomes. Characteristics of the local labor market will therefore be controlled for, mainly by using county indicators such as structure and size of trade and industry, travel distances, unemployment rate according to gender and age group, and unemployment rate to vacant positions ratio.

#### Qualitative material

Firstly, the qualitative analysis will be based upon the key documents that form the basis for the planning, establishment, and running of IPS. This includes public policy documents, formal agreements, plans for the IPS centers, and reports from the organization. The document analysis will be an important part of the initial phase of the project, in order to establish an overview of the formal organization, background, and adaptation of the IPS-intervention. Reports will be collected and analyzed towards the end of the project period, to retain additional information of adaptations and alterations.

Secondly, semi-structured interviews with key informant groups will be conducted. Through the interviews, we will elaborate on the experiences of key persons and their views on barriers and facilitators in carrying out the implementation. Interviews with the various stakeholders will also help to identify the extent of cross-sectorial collaboration between NAV, specialist- and primary care, and its development throughout the project period. The following interviews will be conducted:

1. **Phone interview with key informants** in NAV and the health services, regarding planning, management, and operation of IPS at central and county levels. This will be carried out in the first phase of the project, in order to get a broader idea of the background and foundation of the intervention and the main features at the initial phase. Scope: 5–8 informants. Time: Fall 2014.
2. **Focus groups consisting of staff and management** from all IPS centers. We choose to utilize focus group interviews as a method, as they provide an efficient collection of qualitative data [56]. The strength of focus group approach is also that the form allows for the exchange of experiences in dialogue with others. Time: Fall 2014.
3. **Follow-up phone-interviews with the management of the IPS centers.** The aim is to obtain information about local adaptations and adjustments, as well as specific challenges and barriers that have arisen in the IPS centers during the implementation. Scope: 3 informants. Time: Spring 2015.
4. **Phone interview with collaborators** in NAV and DPS. This will help to identify how intersectoral collaboration has worked, and barriers and facilitators in this area. We will go in depth on cooperative relations between 2–3 selected IPS centers with different target groups of participants. Scope: 10–15 informants. Time: Spring 2015.
5. **In-depth interviews with participants.** The qualitative material will include in-depth interviews with participants, in order to explore their experiences more elaborately. We will perform a purposeful sampling based on relevant characteristics of the target group combined with the responses to the questionnaires. The method of purposeful sampling is chosen in order to get insight into the heterogeneity rather than the frequency of perceived barriers. In-depth interviews will be conducted with participants giving positive versus negative evaluations in the questionnaire. Scope: 12 to 15 informants. Time: Fall 2014.

The 6-month follow-up questionnaires sent to the IPS intervention group will also include a separate set of open-ended questions about the experiences of the intervention, such as accessibility, coordination, and attitudes of service providers, which will be analyzed as part of the qualitative process evaluation.

### Cost/benefit analysis

The third component of the project is an economic assessment of effect as compared to the resources spent on the intervention. IPS is offered in the public sector and financed by community resources, and a standard approach for assessing the profitability of public projects will be used [57].

The cost-benefit analysis will be based on the effect that the intervention has on labor market participation, and the effect that the intervention has on mental health/disability. In the first case, we calculate the economic benefit (measured in Norwegian Kroner, NOK) as a result of the employment effect of the intervention. The calculation compares income and costs of the IPS intervention with income and costs of the control intervention (TAU with AB or APS). In the second case, where the outcome goal represents health benefits as measured by functional improvement or improved quality of life, the costs of the intervention is seen in terms of health gains achieved. Effect may be compared to costs related to different interventions with the same purpose, in order to analyze which intervention provides the greatest health benefit per NOK. Using standardized conversion tools it is also possible to convert health benefits of the interventions to an index of health-related quality of life. This type of analysis also allows for a comparison of gain in quality of life across the different interventions.

The project aims to identify the economic cost and benefit of IPS, in addition to monitoring cost and benefit related to the TAU for the participants in the control intervention group, primarily AB and APS. Both in IPS and AB/APS, income constitutes the present value of the future production gains due to the participants being employed rather than receiving social security benefits. This must be seen in relation to the cost of the respective interventions. We will use a societal perspective, taking into account that the measures affect government budgets, and tax financing influences the adaptation of private actors. We will also take into account that the participants in the IPS intervention group may differ in probability of seeking out specialist in psychiatry in the follow-up period compared to the control intervention group, and we will estimate the costs of such treatment. An observation window of 18 months after inclusion will be used as a basis to assess the extent of treatment/follow-up in the two groups.

The main source of information is register data from NAV and the Norwegian Patient Register. Estimates for the costs associated with different types of treatment must then be calculated based on information about what different types of treatment/services cost, such as appointments with psychiatric specialists or hospitalization days at a psychiatric institution. This must be based both on information about operating expenses at the IPS centers, and pricing of other relevant services within and outside of NAV.

In order to calculate the cost of IPS, we need information on both fixed and variable operating expenses. How this is to be recorded during the project period, for example, if costs are to be registered on the individual level or, as an average per participant based on the annual total expenditure of the IPS centers, will be finalized when this information is available. Since the cost component in the calculations primarily should include additional costs of IPS, costs that would otherwise be incurred in TAU should be subtracted. The details of this must be decided in consultation with NAV and the IPS centers.

### Ethical considerations

The project was submitted to the Norwegian Regional Ethical Committee and therefrom referred to the Norwegian Social Science Data Services, who approved the study (project number: 34989). All principles in the Helsinki declaration will be followed, personal confidentiality is guaranteed, and declarations of voluntary participation with detailed information on the processes will be signed by each participant upon inclusion. The declaration of informed consent emphasizes the right to withdraw from the project at any time without any explanation. We consider the randomization procedure to be ethically acceptable, and so do our collaborators in NAV. The project is registered in the international trial register ClinicalTrials.gov (NCT01964092).

## Conclusion

The effect evaluation of Individual Placement and Support (IPS) is a large and comprehensive project, providing evidence-based information on the important issue of rehabilitating patients who are currently out of the labor market. It will be the first RCT to evaluate the effectiveness of IPS for patients with moderate to severe mental illness within a Norwegian context, and one of the largest RCTs to date investigating the effect of IPS on competitive employment.

## Abbreviations

AB: Work with assistance
APS: Traineeship in a sheltered business
DPS: District Psychiatric Center
IPS: Individual placement and support
NAV: Norwegian labor and welfare administration
RCT: Randomized controlled trial
TAU: Treatment as usual
TMG: Trial management group
TSC: Trial steering committee
